# Appropriateness of Preoperative Antimicrobial Therapy Does Not Impact Operative or 5-Year Mortality for Patients with Infective Endocarditis Requiring Surgical Therapy

**DOI:** 10.1186/1749-8090-10-S1-A100

**Published:** 2015-12-16

**Authors:** John J Squiers, David Xu, Michael A Wait, Michael J DiMaio

**Affiliations:** 1 Department of Cardiovascular and Thoracic Surgery, University of Texas Southwestern Medical Center, Dallas, TX, 75390, USA; 2 Department of Cardiac and Thoracic Surgery, The Heart Hospital Baylor Plano, Plano, TX, 75093, USA

## Background/Introduction

Many factors may potentially affect postoperative outcomes of surgical therapy for infective endocarditis (IE). What effect appropriateness preoperative antimicrobial therapy, as judged by both duration and drug choice, is controversial in the current literature.

## Aims/Objectives

This study examined the characteristics and outcomes of patient with culture-positive IE requiring surgical therapy, with emphasis on the impact on survival of appropriate preoperative antimicrobial therapy.

## Method

Records of 353 consecutive patients undergoing surgery for IE from 1990-2013 were retrospectively reviewed. All patients with definite or probable active IE by modified Duke criteria and with positive blood cultures prior to surgery were included. Two infectious disease clinicians, blinded to patient outcomes, graded appropriateness of preoperative antimicrobial regimens.

## Results

A total of 270 patients (190 men; mean age 46.2 years) met inclusion criteria. Native valves were infected in 219 (81%) patients. Appropriate preoperative antibiotics were administered to 177 (66%) patients. Multi-organism, Enterococcus, and fungal infections were more common in the inappropriately treated group. Recurrent IE was also more common in the inappropriately treated group. Strep viridans infections and IV-drug use were less common in the inappropriately treated group. Otherwise, there were no significant differences in the rates of comorbidities, valve involvement, pathogens, or postop complications (Table). Operative mortality was 12.9% overall, with no significant difference between the appropriately (14%) and inappropriately (11%) treated groups (p = 0.433). Similarly, there was no difference in unadjusted, all-cause mortality between the appropriately (52%) and inappropriately (48%) treated groups (p = 0.545). Mean follow-up time was 3.9 years.

## Discussion/Conclusion

There was no significant difference in operative or 5-year mortality between patients receiving appropriate versus inappropriate preoperative antimicrobial therapy. Thus, surgical treatment seems be of greater importance than appropriate antimicrobial therapy.

